# Supplementary material for: Population Genetics of Phlebotomus papatasi from Endemic and Nonendemic Areas for Zoonotic Cutaneous Leishmaniasis in Morocco, as Revealed by Cytochrome Oxidase Gene Subunit I Sequencing
Source: Microorganisms. 2020 Jul 6;8(7):1010. doi: 10.3390/microorganisms8071010 (PMC7409291; doi:10.3390/microorganisms8071010)
Supplement: Supplementary file 1 [file microorganisms-08-01010-s001.zip › suppdataS1.docx]

| Statistics | Ouarzazate | Non-Ouarzazate | Mean | s.d. |
| --- | --- | --- | --- | --- |
| No. of transitions  No. of transversion  No. of substitutions  No. of indels  No. of ts. Sites  No. of tv. Sites  No. of subst. sites  Total  No. private subst. sites  No. of indel sites  Pi  Theta k  Theta k lower  Theta k upper  Theta H  s. d. Theta H  Theta S  s. d Theta S  Theta pi  s. d Theta pi | 20  2  22  0  20  2  21  33  12  0  5.006  22.054  9.487  53.300  13.619  10.232  5.837  2.276  5.004  2.828 | 19  3  2  0  19  3  21  12  0  4.716  6.553  3.304  12.660  3.487  1.216  4.998  1.785  4.715  2.622 | 19.500  2.500  22.000  0.000  19.500  2.500  21.000  12.000  0.000  4.860  14.303  6.395  32.980  8.553  5.724  5.417  2.031  4.860  2.725 | 0.707  0.707  0.000  0.000  0.707  0.707  0.000  0.000  0.000  0.204  10.960  4.371  28.737  7.164  6.375  0.593  0.347  0.204  0.145 |

**Supp data S1 A:** Molecular diversity as computed with Arlequin

**Supp Data S1 B:** Neutrality test as computed by Arlequin

| Statistics | Ouarzazate | Non-Ouarzazate | Mean | s.d. |
| --- | --- | --- | --- | --- |
| No. of transitions  No. of transversion  No. of substitutions  No. of indels  No. of ts. Sites  No. of tv. Sites  No. of subst. sites  Total  No. private subst. sites  No. of indel sites  Pi  Theta k  Theta k lower  Theta k upper  Theta H  s. d. Theta H  Theta S  s. d Theta S  Theta pi  s. d Theta pi | 20  2  22  0  20  2  21  33  12  0  5.006  22.054  9.487  53.300  13.619  10.232  5.837  2.276  5.004  2.828 | 19  3  2  0  19  3  21  12  0  4.716  6.553  3.304  12.660  3.487  1.216  4.998  1.785  4.715  2.622 | 19.500  2.500  22.000  0.000  19.500  2.500  21.000  12.000  0.000  4.860  14.303  6.395  32.980  8.553  5.724  5.417  2.031  4.860  2.725 | 0.707  0.707  0.000  0.000  0.707  0.707  0.000  0.000  0.000  0.204  10.960  4.371  28.737  7.164  6.375  0.593  0.347  0.204  0.145 |

| Statistics | Ouarzazate | Non-Ouarzazate | Mean | s. d. |
| --- | --- | --- | --- | --- |
| Tajima’s D test  Sample size  S  Pi  Tajima’s D  Tajima’s D p-value  Fu’s FS test  No. alleles (unchecked)  Theta pi  Exp. No. of alleles  FS  FS p-value | 21  21  5.004  -0.537  0.313  15  5.004  8.667  -6.039  0.005 | 38  21  4.715  -0.189  0.484  13  4.715  10.853  -1.110  0.369 | 29.500  21.000  4.860  -0.363  0.398  14.000  4.860  9.760  -3.574  0.187 | 12.028  0.000  0.204  0.246  0.120  14.414  0.204  1.546  3.485  0.257 |
